# Neutrophilic Asthma Is Associated with Increased Airway Bacterial Burden and Disordered Community Composition

**DOI:** 10.1155/2018/9230234

**Published:** 2018-07-09

**Authors:** Xu Yang, Haining Li, Qianli Ma, Qiao Zhang, Changzheng Wang

**Affiliations:** Institute of Respiratory Diseases, Xinqiao Hospital, Army Medical University (Third Military Medical University), Chongqing 400037, China

## Abstract

Neutrophilic asthma (NA) is an important asthma inflammatory phenotype associated with disease severity, airflow limitation, and steroid resistance, and its mechanism is still uncertain. Evidences suggest a potential role for bacteria in its pathogenesis, but, so far, this remains poorly understood. We sought to investigate airway bacterial burden, community composition, and inflammatory response in NA. Fifty-four stable asthmatics without infection were enrolled and separated into either NA group (n = 20) or non-NA group (n = 34). Subject demographics, Asthma Control Test (ACT) scores, medications, and pulmonary functions were documented. Sputum cytology, airway bacterial burden, microbial community composition, and inflammatory cytokines were assessed. The total airway bacterial burden was significantly increased in subjects with NA versus non-NA and was positively correlated with the sputum neutrophil percentage. Airway neutrophilia was associated with less airway bacterial community richness and diversity, along with a distinct community composition. In patients with NA, bacteria in phylum Proteobacteria, especially* Haemophilus* spp. and* Moraxella* spp., showed significant increases in both actual loads and relative abundances, while bacteria in phyla Firmicutes, Actinobacteria, and Saccharibacteria showed decreased relative abundances compared with non-NA. Patients with NA demonstrated higher levels of interleukin-1*β* (IL-1*β*), IL-6, IL-8, IL-12, IL-17A, and tumor necrosis factor-*α* (TNF-*α*) in sputum samples compared with non-NA. Increased bacterial burden and distinct microbiota composition were the key characters of neutrophilic phenotype in asthma, accompanied by excessive airway inflammation. Understanding the relationship between airway microbiota and neutrophilic inflammation may help in treatment and management of asthma, such as targeting airway microbiota.

## 1. Introduction

Asthma is a chronic heterogeneous airway inflammatory disease. According to proportions of sputum eosinophils and neutrophils, asthma is divided into 4 inflammatory subtypes: eosinophilic asthma, neutrophilic asthma (NA), mixed granulocytic asthma, and paucigranulocytic asthma [1]. The pathogenesis of eosinophilic asthma is relatively well elucidated. However, the mechanism driving the neutrophilic phenotype still remains poorly understood.

Although neutrophilic phenotype can be seen across almost all severity of asthma, it seems more common in severe asthma phenotypes [2]. In addition, airway neutrophilia is also linked to asthma exacerbation, airflow limitation, and steroid resistance [3–5]. Therefore, better understanding its characteristics and pathogenesis may be helpful in asthma treatment and management.

In recent years, the lower airway is no longer thought sterile. Patients with asthma have an increased airway bacterial burden and disordered microbiome compared with healthy controls [6, 7]. Furthermore, airway microbiome shows associations with patient's characteristics, disease severity, immune response, and steroid sensitivity [8]. Since both neutrophilic inflammatory phenotype and airway bacterial microbiota are associated with asthma features, there may be a link between them.

In fact, several studies have indicated this possibility. About 60% of patients with NA have pathogenic microorganisms cultured from their bronchoalveolar lavage fluids (BALF) [9]. Endotoxin, component of Gram-negative bacteria, shows elevated levels in airways of NA patients [10]. Potentially pathogenic bacteria, detected by either culture-dependent or culture-independent methods, are observed to be associated with neutrophilic airway inflammation in stable asthmatics [11, 12]. Moreover, using 16S rRNA sequencing, some specific bacterial taxa dominant in certain inflammatory phenotypes are even identified, such as* Streptococcus* spp. in eosinophilic asthma and* Haemophilus influenzae* in NA [13, 14].

However, the patients involved in these studies are mostly focused on or limited to those with severe asthma [13–15], which itself shows a distinct airway microbiome from nonsevere asthma [8]. The relationship between bacteria and asthma inflammatory phenotype entirely is still not well understood. In addition, these studies mostly focused on the relative abundances of microbial communities but ignored showing the differences of the actual communities loads based on total bacterial burden within asthma inflammatory phenotypes. Therefore, the relationship between airway microbiota and inflammatory phenotype needs to be further elucidated.

In the current study, across all spectrum of asthma severity, we investigated the airway bacterial burden and microbial community composition based on inflammatory phenotype stratification and evaluated the inflammatory responses to determine the relationship between airway microbiota and neutrophilic inflammatory phenotype in asthma.

## 2. Materials and Methods

### 2.1. Study Design

Eligible subjects attended an outpatient clinic for a single visit. This cross-sectional study was conducted in accordance with the Declaration of Helsinki and was approved by the Medical Ethics Committee of the Second Affiliated Hospital of the Third Military Medical University. Written informed consent was obtained from all subjects before enrolment in this study. This observational study was registered with the Chinese Clinical Trial Registry (www.chictr.org.cn, Registration No.: ChiCTR-RPC-15007236).

### 2.2. Subjects

All subjects had a diagnosis of asthma according to criteria defined in the 2014 Global Initiative for Asthma (GINA) report. The exclusion criteria for the study included reported asthma exacerbation or use of systemic steroids in the previous 4 weeks, infections or antibiotic use in the previous 4 weeks, smoking more than 5 pack-years or smoking cessation within the last 1 year, and bronchiectasis. To reduce oral contamination, subjects with sputum squamous cell percentages greater than 10% were excluded from the study.

During the visit, subject demographics and medication use were documented. The Asthma Control Test (ACT) was used to assess patient-reported symptoms. Spirometry was performed according to the American Thoracic Society/European Respiratory Society (ATS/ERS) guidelines [16]. Induced sputum was used to evaluate inflammatory phenotypes and analyze the airway bacterial burden and microbiome. Eligible subjects were categorized into either the NA or non-NA group, based on a sputum neutrophil cut-off value of 65% [17]. Subjects with NA were those who had increased sputum neutrophil proportions (>65%), and the others were subjects with non-NA.

### 2.3. Sputum Processing and DNA Extraction

Induced sputum was collected and processed within 1 hour, as previously described [7]. Briefly, induced sputum was obtained by having subjects inhale ultrasonically nebulized 3% saline after 400 *μ*g salbutamol inhalation. Saliva was spit out and sputum was coughed into a separate clean cup. Plugs were carefully selected and dispersed using dithiothreitol at 37°C. Total cell counts and viability were performed with a haemocytometer and trypan blue. A differential cell count of leukocytes was obtained by counting at least 400 cells on a slide stained with Wright-Giemsa. Total bacterial genomic DNA was extracted from sputum supernatants with the TIANamp Bacteria DNA Kit (TIANGEN, Beijing, China), according to the manufacturer's instructions. After measuring the concentration and purity with NanoDrop ND2000 (Thermo, USA), the DNA was stored at −20°C until use. The remaining supernatants from sputum samples were stored at −80°C for cytokine measurements.

### 2.4. Bacterial Burden and Sequencing

For total bacterial burden assessment, a standard substance and a standard curve were first made. The V3-V5 region of 16S rRNA gene was amplified by using the forward primer 357F (5′-CTCCTACGGGAGGCAGCAG-3′) and the reverse primer 926R (5′-CCGTCAATTCCTTTRAGTTT-3′). Each 15 *μ*l PCR reaction contained 7.5 *μ*l of 2 × Taq PCR Master Mix (TIANGEN, Beijing, China), 0.6 *μ*l of 20-*μ*M each primer, 1 *μ*l of template DNA, and 5.3 *μ*l of PCR-grade water. Thermal cycling conditions were as follows: initial denaturation at 94°C for 3 minutes and 25 cycles of 94°C for 30 seconds, 57°C for 30 seconds, and 72°C for 30 seconds. After being pooled, separated on 1.5% agarose gels containing ethidium bromide (EB), excised, and purified, the PCR products were cloned into the pGM-T Vector (TIANGEN, Beijing, China) according to the manufacturer's instructions. The ligation product was transferred into DH5*α* cells; after enrichment, DH5*α* cells containing the ligation product were identified. The ligation product (the standard substance) was extracted, concentrated as 80.77 ng/*μ*l, sequenced as 591 bp by using T7 and SP6 primers (5′-TAATACGACTCACTATAGGGCGA-3′ and 5′-ATTTAGGTGACACTATAGAA-3′), and calculated as containing 2.043 × 10^10^ 16S rRNA gene copies/*μ*l. By amplifying serial dilutions of the plasmid DNA, a standard curve was established. We performed 20 *μ*l PCR reactions in triplicate with 10 *μ*l of 2 × PCR Master Mix (QuantiNova SYBR Green PCR Kit, Qiagen), 0.5 *μ*l of 20-*μ*M 357F primer solution and 0.5 *μ*l of 20-*μ*M 926R primer solution, 1 *μ*l of template DNA, and 8 *μ*l of PCR-grade water. PCR cycling conditions were as follows: initial denaturation at 95°C for 2 minutes and 40 cycles of 95°C for 5 seconds and 61°C for 35 seconds. The extracted bacterial genomic DNA from the sputum was amplified simultaneously. 16S rRNA gene copy numbers per *μ*l in the extracted DNA were calculated against the standard curve. Bacterial burden was further determined based on volume of DNA and mass of sputum.

For 16S rRNA gene sequencing, we amplified the V3-V4 region of 16S rRNA gene in extracted DNA by using the forward primer 338F (5′-ACTCCTACGGGAGGCAGCA-3′) and the reverse primer 806R (5′-GGACTACHVGGGTWTCTAAT-3′). The PCRs were carried out in triplicate with 4 *μ*l of a 10 × Buffer, 2 *μ*l of 2.5-mM dNTPs solution, 0.8 *μ*l of 5-*μ*M each primer, 0.2 *μ*l of rTaq, 0.2 *μ*l of BSA, 10 ng of template DNA, and PCR-grade water to bring the reaction volume to 20 *μ*l. PCR cycling conditions were as follows: 95°C for 3 minutes and 27 cycles of 95°C for 30 seconds, 55°C for 30 seconds, and 72°C for 45 seconds; a final extension was run at 72°C for 10 minutes. Triplicate PCR products were pooled and purified. Paired-end reads were generated on an Illumina MiSeq PE250 (Majorbio Bio-Pharm Technology Co., Ltd., Shanghai, China).

### 2.5. Cytokine Measurements

Cytokines were measured in sputum supernatants using a Luminex bead-based assay (Merck Chemicals Co., Ltd., Shanghai, China), which was performed according to the manufacturer's instructions (Human High Sensitivity T Cell Panel, Millipore Corporation). Mean fluorescent intensity data were analyzed, and cytokine concentrations were calculated using a 5-parameter logistic or spline curve-fitting method on a MAGPIX 12157001 system with xPONENT software.

### 2.6. Statistical Analyses

To identify bacterial communities, reads that did not match the barcode sequences were filtered out. The remaining reads were clustered into operational taxonomic units (OTUs) with 97% similarity using Usearch (version 7.1). The representative 16S rRNA gene sequence of each OTU was analyzed by the RDP Classifier [18] (version 2.2) using the Silva 16S rRNA database [19] (release 119) with a 70% confidence threshold. The species richness estimators (Chao and ACE) and diversity indices (Shannon and Simpson) were obtained using Mothur [20] (version v.1.30.1). *β*-Diversity was determined by nonmetric multidimensional scaling (NMDS) based on Bray Curtis distance, and a permutational multiple ANOVA (PERMANOVA) test (Adonis) was calculated by using R software (version: 3.3.3, vegan package).

Other statistical analyses were performed using SPSS (IBM, version 22.0). Continuous data were tested for normality by Kolmogorov-Smirnov test. The mean and standard deviation (SD) for each group were computed for variables fitting a normal distribution, including age, body mass index (BMI), percent predicted forced expiratory volume in 1 second (FEV1), percent FEV1/forced vital capacity (FVC), neutrophils percentage, macrophage percentage, and relative abundances of Proteobacteria, Firmicutes, Bacteroidetes, and Fusobacteria. An independent-sample* t*-test was performed to compare differences of these variables between groups. The median and first and third quartiles were computed for variables that were not normally distributed, including ACT score, inhaled corticosteroids (ICS) daily dose, total and differential cell counts in sputum, bacterial burden, community richness and diversity estimators, relative abundances, and actual loads of most bacterial taxa and inflammatory cytokines. The Mann–Whitney* U* test was performed to compare the distributions of these variables between groups. Fisher's exact probability test was performed to analyze categorical variable, sex. Spearman's correlation coefficients were calculated for the correlations between neutrophil percentage and total bacterial burden, community richness and diversity, relative abundances, and actual loads of bacterial taxa and inflammatory cytokines.

## 3. Results

### 3.1. Subjects' Characteristics

Fifty-four eligible subjects were enrolled in this study. Of these, 20 subjects had NA, and 34 subjects had non-NA (**Table 1**). Subjects with NA had similar age, sex distribution, BMI, ICS daily dose, ACT score, pulmonary function, and squamous cell percentage in sputum compared with non-NA subjects. However, sputum inflammatory cells in the NA group showed significantly higher viability than those in the non-NA group, with an increased number of neutrophils and a decreased number of eosinophils and macrophages (**Table 1**).

### 3.2. Total Airway Bacterial Burden

**Figure 1:**
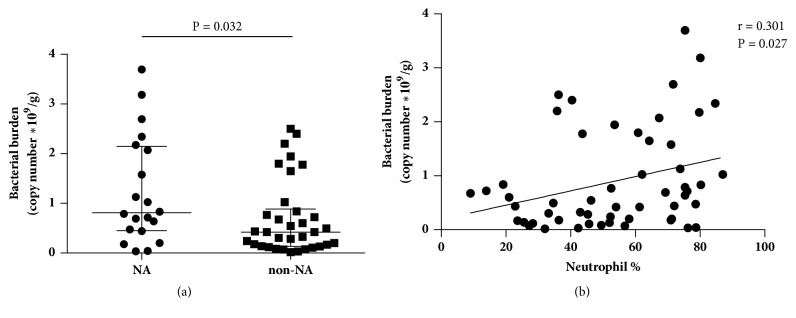
**Bacterial burden in sputum samples from subjects with NA and non-NA.** Airway bacterial burden was evaluated based on 16S rRNA gene copy number in sputum samples from 20 subjects with NA and 34 subjects with non-NA. Comparison between groups was made by using Mann–Whitney* U* test (a). Correlation coefficient between bacterial burden and neutrophil percentage was calculated by Spearman's correlation (b). Data are presented as median and the interquartile range. The dots represent all of the values. NA: neutrophilic asthma.

Total airway bacterial burden was increased in subjects with NA compared with that in subjects with non-NA (median, 0.81 × 10^9^/g versus 0.42 × 10^9^/g, P = 0.032;**Figure 1(a)**). Total airway bacterial burden showed positive correlation with sputum neutrophil percentage (r = 0.301, P = 0.027;**Figure 1(b)**).

By using the bivariate correlation, total airway bacterial burden showed negative correlations with both eosinophil % (r = -0.296, P = 0.030) and macrophage % (r = -0.297, P = 0.029). However, these two variables were both negatively correlated with neutrophil % (r = -0.433, P = 0.001 and r = -0.764, P < 0.001, resp.). By using partial correlation (control for neutrophil %), no significant correlations between total bacterial burden and eosinophil % or macrophage % were found. In addition, the counts of eosinophil and macrophage showed no significant correlations with total bacterial burden either, while neutrophil count was positively correlated with total bacterial burden (r = 0.300, P = 0.027).

### 3.3. *α*- and *β*-Diversity

**Figure 2:**
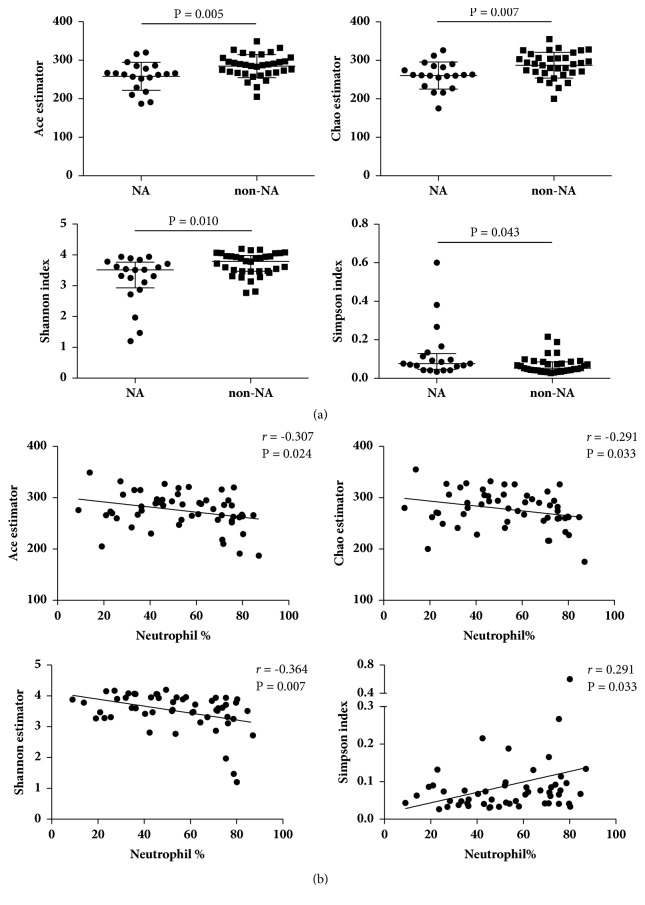
**Bacterial community richness and diversity in sputum samples from subjects with NA and non-NA.** Community richness was evaluated by Ace estimator and Chao estimator, and community diversity was evaluated by Shannon index and Simpson index. Comparison of community richness between groups was made by using* t*-test, while comparison of community diversity between groups was made by using Mann–Whitney* U* test (a). Correlation coefficients between these indices and neutrophil percentage were calculated by Spearman's correlation (b). Data are presented as mean and SD or median and the interquartile range, respectively. The dots represent all of the values. NA: neutrophilic asthma.

Neutrophilic asthmatics demonstrated less airway bacterial community richness than nonneutrophilic asthmatics, indicated by significantly lower Ace estimator and Chao estimator in NA subjects (P = 0.005 and P = 0.007, resp.). Bacterial community diversity was also decreased in neutrophilic asthmatics, suggested by significantly lower Shannon index and higher Simpson index (P = 0.010 and P = 0.043, resp.) (**Figure 2(a)**). Simpson index showed a positive correlation with the sputum neutrophil percentage, while the other three indices showed negative correlations (P < 0.05) (**Figure 2(b)**).

**Figure 3:**
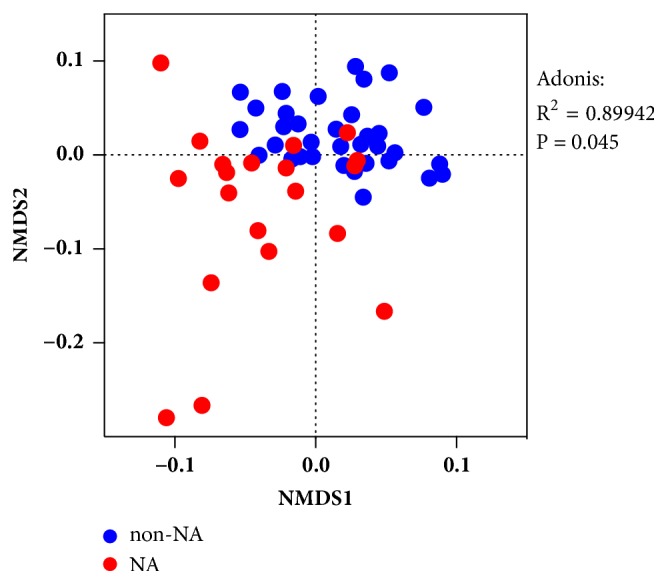
**Bacterial community distributions in sputum samples from subjects with NA and non-NA.**  *β*-Diversity grouped by sputum neutrophil percentage was evaluated by NMDS based on Bray Curtis distance. Comparison between two groups was made by using a PERMANOVA test (Adonis). Each dot represents the overall bacterial community in each sample. NA: neutrophilic asthma; NMDS: nonmetric multidimensional scaling.

NMDS based on Bray Curtis distance showed that the overall microbiota distributions of sputum samples in NA were distinguished from those in non-NA (**Figure 3**). A PERMANOVA test (Adonis) indicated a consistent finding with the observation and demonstrated that sputum neutrophil percentage significantly contributed to the difference of the airway bacterial community composition (R^2^ = 0.89942, P = 0.045).

Collectively, these results indicated a significant relationship between airway bacterial community composition and airway neutrophilia.

### 3.4. Community Composition

Phylogenetic analysis identified 530 bacterial OTUs, representing 19 phyla and 240 genera. However, many of them were relatively uncommon with very little abundances; to analyze and detect associations with these uncommon OTUs would not make a lot of sense. Therefore, we focused analyses on the OTUs with sequences* ⩾*1% of the total.

**Figure 4:**
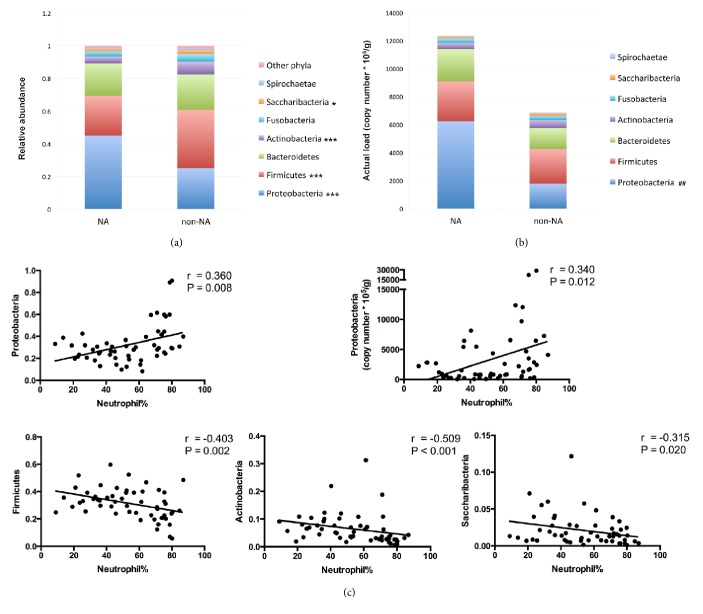
**Bacterial phyla in sputum samples from subjects with NA and non-NA.** In comparisons of relative abundances (a), differences of Proteobacteria, Firmicutes, Bacteroidetes, and Fusobacteria between groups were determined by using* t*-test due to their normal distributions, while differences of the other phyla were determined by using Mann–Whiney* U* test. In comparisons of actual loads (b), differences of all phyla were determined by using Mann–Whitney* U* test. Correlation coefficients between bacterial phyla and neutrophil percentage were calculated by Spearman's correlation (c). Significant differences between relative abundances of phyla were shown as ^*∗*^P < 0.05 and ^*∗∗∗*^P < 0.001. Significant differences between actual loads of phyla were shown as ^##^P < 0.01. The dots represent all of the values. NA: neutrophilic asthma.

At the level of phylum, these OTUs belonged to the phyla Proteobacteria (32.4%), Firmicutes (31.4%), Bacteroidetes (21.1%), Actinobacteria (6.5%), Fusobacteria (4.1%), Saccharibacteria (2.1%), and Spirochaetae (1.0%). Among them, Proteobacteria were present in higher proportions in neutrophilic asthmatics (mean, 45.0% versus 25%; P < 0.001). In contrast, Firmicutes (mean, 24.3% versus 35.6%; P < 0.001), Actinobacteria (median, 2.9% versus 7%; P < 0.001), and Saccharibacteria (median, 1.3% versus 1.6%; P = 0.047) were found less frequently in subjects with NA than non-NA (**Figure 4(a)**). However, from the aspect of actual loads of taxa, only bacteria from the phyla Proteobacteria (median, 3170.7 × 10^5^/g versus 790.8 × 10^5^/g; P = 0.004) showed significant increase in its actual load in the NA group (**Figure 4(b)**). Sputum neutrophil percentage showed positive correlations with the relative abundance and actual load of Proteobacteria and negative correlations with the relative abundances of Firmicutes, Actinobacteria, and Saccharibacteria (P < 0.05,**Figure 4(c)**).

**Figure 5:**
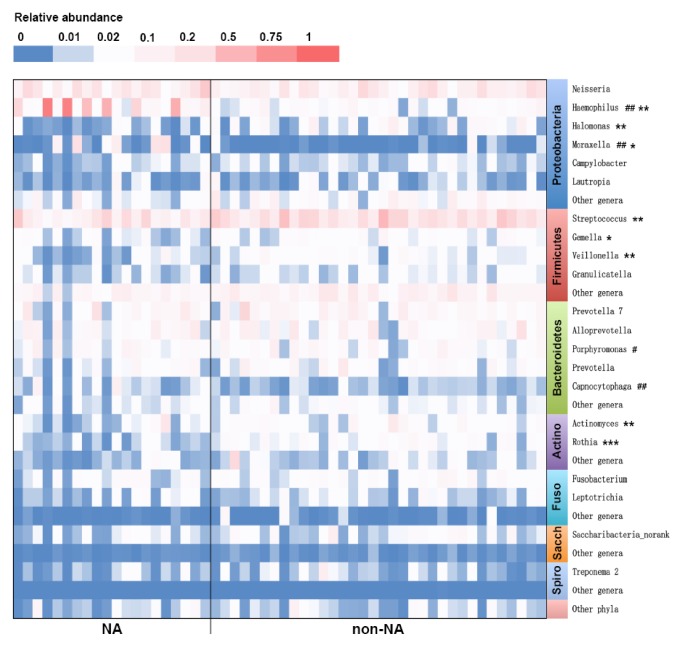
**Heat map showing the relative abundances of OTUs in sputum samples from subjects with NA and non-NA.** Comparisons between groups were made by using Mann–Whitney* U* test. Significant differences of relative abundances between groups were shown as ^*∗*^P < 0.05, ^*∗∗*^P < 0.01, and ^*∗∗∗*^P < 0.001. Significant differences of actual loads between groups were shown as ^#^P < 0.05 and ^##^P < 0.01. NA: neutrophilic asthma; Actino: Actinobacteria; Fuso: Fusobacteria; Sacch: Saccharibacteria; Spiro: Spirochaetae.

A total of 21 genera were represented by* ⩾*1% of the total number of sequences. Among them, four potential pathogens,* Haemophilus* spp. (phylum Proteobacteria),* Porphyromonas *spp. (phylum Bacteroidetes),* Moraxella* spp. (phylum Proteobacteria), and* Capnocytophaga *spp. (phylum Bacteroidetes), showed significant increases in their actual loads in samples from subjects with NA compared with non-NA (P < 0.05).* Haemophilus *spp. and* Moraxella* spp. showed very striking increases in actual loads that there were significantly higher relative abundances of them. In the patients with NA, 13 of 20 samples (65%) had a total relative abundance of* Haemophilus *spp. and* Moraxella* spp. higher than 10%, compared with 0 of 34 samples (0%) in the patients with non-NA (P < 0.001,**Figure 5**). Meanwhile, proportions of 6 genera,* Streptococcus* spp.,* Actinomyces* spp.,* Halomonas* spp.,* Gemella* spp.,* Rothia* spp., and* Veillonella* spp., were significantly less abundant in subjects with NA compared with non-NA (P < 0.05,**Figure 5**).

### 3.5. Inflammatory Response

Increased airway bacterial burden and the disordered community composition in NA might promote an excessive inflammatory response. To evaluate the airway inflammation, we assessed the levels of several cytokines in the supernatant of sputum samples. Subjects with NA demonstrated higher levels of interleukin-1*β* (IL-1*β*), IL-6, IL-8, IL-12, IL-17A, and tumor necrosis factor (TNF)-*α* in sputum samples compared with non-NA subjects (**Table 2**). These cytokines all revealed significant positive correlations with neutrophil percentage in sputum (**Table 2**).

## 4. Discussion

In the current study, we evaluated the total bacterial burden, bacterial community composition, and inflammatory response based on inflammatory phenotype in stable asthma. Compared with patients with non-NA, neutrophilic asthmatics demonstrated greater total bacterial burden in airways, along with a distinct airway microbiome and excessive inflammation. These results highlight the relationship between airway bacterial microbiota and neutrophilic inflammation, suggesting a potential therapeutic target for treatment and management of NA.

Total airway bacterial burden is reported to be greater in asthmatics than in healthy controls [6]. Here, we extended this to show that it is even greater in patients with NA than non-NA, with a positive correlation with sputum neutrophil percentages. Community richness and diversity demonstrated reduced levels in patients with NA and revealed significantly negative correlations with airway neutrophilia. Analysis of *β*-diversity also showed a marked difference in the distribution of bacterial microbiota between samples from NA and non-NA. An earlier study focused on patients with severe asthma also identified sputum neutrophil percentage as the strongest predictor of microbiota variance [15]. These results suggest that great bacterial burden and disordered microbial composition were key characters in airway neutrophilic phenotype in asthma.

Further looking inside the community composition, we found that phylum Proteobacteria, which represens lots of pathogens, had a significantly increased relative abundance in patients with NA compared with non-NA. Meanwhile, phyla Firmicutes, Actinobacteria, and Saccharibacteria, particular* Streptococcus* spp.,* Gemella* spp.,* Veillonella* spp.,* Actinomyces* spp., and* Rothia* spp. showed reduced abundances in NA. These findings were consistent with previous report in poorly controlled asthma [14]. In addition, compared with noneosinophilic asthma, Firmicutes/Streptococcus and Actinomycetaceae were recently reported to be abundant in and positively associated with eosinophilic asthma [13, 21]. This study partly suggested the inverse relationship between Firmicutes/Streptococcus and Actinobacteria abundance and NA, consistent with what was shown in our study, as neutrophilic asthmatics presented significantly decreased eosinophils. Furthermore, relative abundances of these phyla all showed significant correlations with sputum neutrophil percentage, further supporting the opinion that there is substantial association between airway neutrophilia and airway microbiota dysbiosis. However, from the aspect of actual loads of taxa, only Proteobacteria revealed a significant difference. This indicated that, in NA, decreases in relative abundances of some taxa from the other phyla were mostly caused by the great increase of Proteobacteria, especially* Haemophilus* spp. and* Moraxella* spp., rather than by decreases of the actual loads of themselves.

Accompanied by the greater bacterial burden and disordered composition, excessive airway inflammation was observed in NA than non-NA. Although the cause and effect between airway neutrophilic inflammation and microbiome dysbiosis cannot be determined because of the limitation of cross-sectional studies, they may be interdependent. On one hand, airway inflammatory response may put selective pressure on airway microbiota. Patients with asthma had much greater abundances of Proteobacteria and* Haemophilus* spp. than nonasthmatics [7, 22], suggesting that allergy itself may promote the susceptibility of colonization of these bacteria in the lower airway, while airway neutrophilia were demonstrated to further promote the overgrowth of these taxa in the current study. On the other hand, predominance of Proteobacteria/*Haemophilus* spp. in airway microbiota in NA may interpret the excessive inflammatory response accompanied by increased airway neutrophilia compared with non-NA. Given the roles of neutrophils in innate immunity, they will be activated and recruited into airways to fight against invading pathogens [23], resulting in excessive neutrophilic inflammation. Significant increases in IL-1*β* and IL-6 levels in NA versus non-NA suggested the possibility of increased differentiation of T helper (Th) 17 cells [24]. The elevation of IL-17 level in NA compared with non-NA further indicated this. In addition to IL-17, IL-8, IL-6, and TNF-*α* may also be the effective factors produced by Th17 cells and promote neutrophil recruitment and activation, leading to airway neutrophilic inflammation [25, 26]. Compared with other common pathogens, such as* Streptococcus pneumonia* and* Moraxella catarrhalis*,* Haemophilus influenzae*, which significantly increased in NA, is associated with greater airway inflammatory response [27]. In addition,* Haemophilus influenzae* promotes an IL-17 mediated immune response and induces neutrophilic inflammation in a murine model of asthma [28]. These suggest that increased airway bacterial burden, especially* Haemophilus influenzae*, promotes a Th17/IL-17 associated airway neutrophilic inflammation in NA.

However, although increased Th17/IL-17 helps to eliminate bacteria, it may also contribute to steroid resistance [29, 30]. In fact,* Haemophilus influenzae* is the predominant pathogen involved in steroid resistance and associated with persistent wheezing [31, 32]. Among the patients with steroid resistance, predominance of* Haemophilus* spp. and* Moraxella catarrhalis* in the airway is associated with impaired lung function and higher airway neutrophil numbers [12]. Proteobacteria, which contains* Haemophilus influenzae*,* Moraxella catarrhalis*, and many other pathogens, is also associated with Th17-associated gene expression and poor asthma control [8]. These suggest that the increased total airway bacterial burden and the disordered airway microbial community composition may be the potential links between NA and treatment-resistant severe asthma. For these patients, airway microbiota can be a potentially therapeutic target.

There are some potential confounding factors that needed to be considered. First, sputum sample is potentially contaminated by oral flora during the process of obtaining. In the current study, to minimize the potential contamination, all samples with squamous cell > 10% were excluded. Patients enrolled had low and similar sputum squamous cell percentages between groups. In addition, the airway microbiome we found in this study is distinct from that resident in the upper airway or oral cavity [33, 34]. These indicated that effect of oral contamination is negligible. Second, increased neutrophil number and total airway bacterial burden can be associated with respiratory tract infections. The treatment of infection, antibiotic therapy, may kill bacteria broadly or selectively. Therefore, airway bacterial burden and microbiota composition may be misleading. Although we excluded all patients who reported features of respiratory infection or taking antibiotic treatment in the previous 4 weeks to avoid these effects, some residual effects might exist in some subjects with infection or earlier antibiotic exposure. Third, corticosteroid therapy may affect the levels of neutrophils in airways and may even lead to alterations in airway and lung microbiome [35, 36]. In our study, current ICS doses were similar between the two groups; therefore, increase in Proteobacteria, especially* Haemophilus* spp., in NA is primarily associated with neutrophilic phenotype. However, the relative poor response to corticosteroids in NA patients may lead a treatment with potentially higher dose of corticosteroids and mislead the observation of airway microbiome dysbiosis; effects of corticosteroids still needed to be noted.

In this cross-sectional study, neutrophilic phenotype in asthma is associated with a great airway bacterial burden, along with an excessive airway inflammation. However, the sputum inflammatory phenotype is reported to be unstable [37, 38]. The percentage of subjects with a stable neutrophilic phenotype is even smaller [39]. Whether the variation of inflammatory phenotype is related to variation of airway bacterial burden is still uncertain. A longitudinal observation is required to elucidate that. The persistence of a high burden of bacterial microbiota in airway, especially Proteobacteria, may worsen clinical outcomes through its effects persistence. To further highlight the relationship between neutrophilic inflammation, airway microbiome, and steroid-resistant severe asthma, study on the effect of chronic bacterial colonization on the pathogenesis and inflammatory phenotype of asthma is also required.

## 5. Conclusions

This study demonstrated that neutrophilic inflammatory phenotype in asthma is associated with increased bacterial burden in airways, along with a disordered airway microbiome and excessive airway inflammation. Airway dysbiosis is one of the key factors underlying the heterogeneity of asthma inflammatory phenotypes. It promotes an understanding of the relationship between airway neutrophilia and bacterial microbiota and may help in the treatment and management of asthma.

## Figures and Tables

**Table 1:** **Characteristics of subjects**.

Characteristics	NA (n = 20)	Non-NA (n = 34)	P
Age, y	47.4±10.5	42.9±11.3	0.156^*∗*^
Sex, male (%)	10 (50)	10 (29.4)	0.154^&^
BMI, kg/m^2^	24.0±3.20	24.4±3.7	0.649^*∗*^
ICS dose, *μ*g/d	500 (200, 500)	250 (200, 500)	0.193^#^
ACT score	23 (22, 24)	22 (21, 24)	0.425^#^
FEV1% predicted	72.0±18.9	75.0±13.5	0.497^*∗*^
FEV1/FVC%	66.2±10.6	69.1±8.8	0.277^*∗*^
Squamous cells %	2.3 (1.1, 4.8)	2.1 (1.2, 5.8)	0.713^#^
Viability %	89.2 (86.0, 92.5)	82.8 (77.3, 87.2)	0.008^#^
Total cell count ×10^6^/g	14.0 (7.8, 17.5)	10.0 (6.7, 15.1)	0.325^#^
Neutrophils %	75.8±5.1	40.7±14.9	<0.001^*∗*^
Neutrophils count ×10^6^/g	10.7 (5.7, 13.8)	4.0 (2.2, 7.6)	<0.001^#^
Eosinophils %	1.1 (0.2, 3.4)	3.1 (0.9, 17.1)	0.016^#^
Eosinophils count ×10^6^/g	0.1 (0.0, 0.3)	0.2 (0.1, 1.9)	0.053^#^
Macrophages %	15.8±5.6	41.9±17.5	<0.001^*∗*^
Macrophages count ×10^6^/g	1.9 (1.3, 2.6)	3.9 (2.3, 6.4)	<0.001^#^
Lymphocytes %	4.6 (3.5, 9.8)	5.4 (3.1, 9.8)	0.886^#^
Lymphocytes count ×10^6^/g	0.7 (0.3, 1.4)	0.5 (0.2, 1.3)	0.038^#^

Data are presented as n, n (%), mean ± SD, and median (quartile 1, quartile 3) unless otherwise indicated. The ICS dose is presented in fluticasone equivalents.

NA: neutrophilic asthma; BMI: body mass index; ICS: inhaled corticosteroids; ACT: Asthma Control Test; FEV1: forced expiratory volume in 1 second; FVC: forced vital capacity; SD: standard deviation.

^*∗*^ *t*-test.

^#^Mann–Whitney *U* test.

^&^Fisher's exact test.

**Table 2:** **Cytokines in supernatant of sputum samples from subjects with NA and non-NA**.

Cytokines	Comparison between groups			Spearman's correlation	
	NA	Non-NA	P	Rho	P
IL-1*β*, pg/ml	515.4 (226.4, 1523.9)	192.1 (69.3, 527.1)	0.010	0.511	<0.001
IL-6, pg/ml	1410.5 (633.6, 3395.1)	419.7 (290.6, 1312.8)	0.031	0.277	0.042
IL-8, mg/ml	49.9 (28.1, 54.4)	28.7 (6.9, 48.1)	0.015	0.476	<0.001
IL-12, pg/ml	2.3 (2.0, 4.3)	1.5 (1.1, 2.2)	0.002	0.541	<0.001
IL-17A, pg/ml	4.3 (2.2, 10.8)	2.9 (1.4, 4.1)	0.021	0.513	<0.001
TNF-*α*, pg/ml	313.5 (119.0, 1046.3)	180.1 (90.5, 317.9)	0.032	0.454	0.001

Data are presented as median (quartile 1, quartile 3) unless otherwise indicated. Significant differences between groups were determined by using Mann–Whitney *U* test. Correlation coefficients between cytokines and neutrophil percentage were calculated by Spearman's correlation.

NA: neutrophilic asthma; IL: interleukin; TNF: tumor necrosis factor.

## Data Availability

The data used to support the findings of this study are available from the corresponding author upon request.
